# Efficacy and Safety of Pembrolizumab Monotherapy for Recurrent/Unresectable/Metastatic Oral Squamous Cell Carcinoma: A Single-Center Study in China

**DOI:** 10.1155/2022/7283946

**Published:** 2022-08-28

**Authors:** Jieying Li, Zongxuan He, Yueqin Tao, Xiaochen Yang, Shengyou Ge, Haoyue Xu, Wei Shang, Kai Song

**Affiliations:** ^1^Department of Oral and Maxillofacial Surgery, The Affiliated Hospital of Qingdao University, Qingdao, Shandong Province, China; ^2^School of Stomatology, Qingdao University, Qingdao, Shandong Province, China

## Abstract

**Background:**

Although pembrolizumab is recommended as a first-line treatment for advanced recurrent/unresectable/metastatic (R/U/M) head and neck squamous carcinoma, the differences in its efficacy among different populations need to be investigated.

**Methods:**

We reviewed 15 consecutive patients with R/U/M oral squamous cell carcinoma (OSCC) treated with pembrolizumab monotherapy at the Affiliated Hospital of Qingdao University between February 2021 and May 2022. All the 15 patients had known programmed death-ligand 1 expression and received multiple cycles of pembrolizumab monotherapy as first-line treatment. We evaluated and analyzed patients' basic characteristics, time to first remission, the clinical efficacy of pembrolizumab monotherapy, and treatment-related adverse reactions.

**Results:**

The objective response rate of the 15 patients was 60%. Six patients (40.0%) achieved partial response, while three patients (20.0%) achieved complete response. In our study, the objective response status of the patients was observed in two to five cycles (mean, 3.6 cycles). For patients who responded well to immunotherapy, the mean Karnofsky Performance Status (KPS) score after treatment was significantly higher than that before treatment (*P* < 0.001). The progression-free survival rates were 66.9% and 50.1% at 6 months and 1 year, respectively. Eight adverse events were observed, comprising four cases of rash and one case each of hypothyroidism, interstitial pneumonia, cheilitis, and cerebral thrombosis.

**Conclusion:**

Our study suggests that pembrolizumab is beneficial to the most responsive patients with R/U/M OSCC in our single-center study and may shed light on the management of OSCC.

## 1. Introduction

Oral squamous cell carcinoma (OSCC) is a common subtype of head and neck squamous cell carcinoma (HNSCC), with a 5-year survival rate of approximately 50% [1, 2]. In the past decade, cetuximab plus platinum-fluorouracil chemotherapy has been the primary first-line treatment option for recurrent or metastatic OSCC as it helps in local control and improves overall survival in some patients; however, the overall prognosis of patients with advanced OSCC remains poor [3–6]. Therefore, for patients with advanced recurrent/unresectable/metastatic (R/U/M) OSCC, prolonging life expectancy and improving quality of life remains challenging for oncologists.

Immunotherapy has caused a paradigm shift in cancer treatment. In particular, immune checkpoint inhibitors targeting programmed cell death 1 (PD-1) have been effective in treating certain cancer types. Presently, immunotherapy has shown good efficacy for more than 10 solid tumors, including melanoma and lung cancer [7, 8]. A KEYNOTE-024 randomized controlled trial (pembrolizumab) conducted on patients with metastatic non-small-cell lung cancer indicated that the 5-year survival rate of patients treated with pembrolizumab significantly improved from 16.3% to 31.9% compared to those subjected to platinum-based chemotherapy [9].

Recently, the clinical benefits of immunotherapy in patients with HNSCC have been reported. In a KEYNOTE-048 prospective randomized controlled study on patients expressing programmed death-ligand 1 (PD-L1), pembrolizumab monotherapy or a combination of chemotherapy was reportedly superior to the EXTREME regimen in terms of meaningful improvements in overall survival (OS), progression-free survival (PFS), and objective response rate (ORR) [10]. According to the 2021 National Comprehensive Cancer Network guidelines for Head and Neck Cancer (published on November 9, 2020), pembrolizumab was first proposed as the first-line treatment for advanced R/U/M HNSCC [11]. Subsequently, pembrolizumab monotherapy was approved by the National Medicine Products Administration (NMPA) of China for treating patients with advanced R/U/M HNSCC and PD-L1 combined positive score (CPS) ≥ 20. Although immunotherapy has revolutionized HNSCC treatment, the efficacy and safety of pembrolizumab varies by geographic region and ethnicity [10, 12]. Moreover, limited data are available on pembrolizumab efficacy in the Chinese population with HNSCC, especially OSCC. In the present study, we evaluated 15 patients with advanced R/U/M OSCC who were treated with pembrolizumab to evaluate its antitumor efficacy and safety among the Chinese OSCC population. We hope that the results of our study will be a useful reference for the immunotherapy of patients with advanced OSCC.

## 2. Materials and Methods

### 2.1. Patients and Treatments

Based on the NCCN guidelines (Version 1.2021), the inclusion criterion of R/U/M was summarized as follows: R, loco-regional recurrence (recurrence of the primary tumor or the draining lymph nodes) or persistent disease; U, newly diagnosed T4b, N0–3, M0, or unresectable nodal disease, or unfit for surgery; M, distant metastases [11]. In our study, we reviewed 15 consecutive patients with R/U/M OSCC who were treated with pembrolizumab monotherapy at Affiliated Hospital of Qingdao University between February 2021 and May 2022. All the patients were histopathologically confirmed to have OSCC and tested positive for PD-L1 expression based on CPS testing of HNSCC [13]. All the patients received first-line pembrolizumab monotherapy (200 mg) intravenously every 3 weeks [10, 14]. The treatment regimen was re-evaluated when any of the following issues were noted: grade 4-5 adverse reactions (AEs), progressive disease, or no positive response by the fifth cycle. In addition, 20 patients with no surgery or radiotherapy option received the conventional chemotherapy regime (platinum and 5-fluorouracil or paclitaxel) with or without cetuximab in the CPS of 1 or more populations. These populations with chemotherapy were used as the control group for the evaluation of PFS without pembrolizumab in our study. All the patients were followed up until the end of the study (May 1, 2022). This study was approved by the review board of the Affiliated Hospital of Qingdao University and conducted in accordance with the Declaration of Helsinki (1964). This manuscript is available as a preprint at https://www.researchsquare.com/article/rs-1708624/v1 [15].

### 2.2. Data Collection and Evaluation

Patients' demographic and clinical data, medical history, PD-L1 expression, and Karnofsky performance status (KPS) score were obtained [16]. Follow up was conducted regularly by telephone calls or during clinic visits. Patients' quality of life (QoL) was evaluated using the KPS scale, which had a maximum score of 100; the higher the score, the better the health status of the patient [16]. Response to pembrolizumab monotherapy was assessed by regular imaging examination and observation of objective tumor response according to the suggestions of the multidisciplinary team (MDT) and Response Evaluation Criteria in Solid Tumors version 1.1 (RECIST 1.1) [17]. Data were collected from the initiation of pembrolizumab monotherapy to the end of our study on May 1, 2022. Immune-related adverse events (irAEs) were evaluated according to the Common Terminology Criteria for Adverse Events, version 5.0 [18].

### 2.3. Data Analysis

A paired *t*-test was conducted to compare the KPS scores before and after pembrolizumab monotherapy [19]. PFS was determined using the Kaplan–Meier method. Data were analyzed using the SPSS version 25.0 (International Business Machines Corp., Chicago, IL, USA). The results with *P* < 0.05 were considered statistically significant.

## 3. Results

### 3.1. Demographic and Clinical Data of Patients

**Figure 1:**
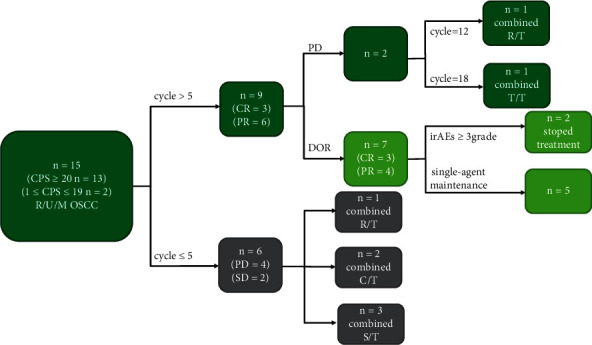
Treatment schema roadmap of all patients in our study. The efficacy of monotherapy in patients with OSCC was assessed according to Response Evaluation Criteria in Solid Tumors version 1.1. CR = complete response; PR = partial response; SD = stable disease; PD = progressive disease; DOR = duration of response; irAEs = immune-related adverse events; *C*/*T* = chemotherapy; *R*/*T* = radiotherapy; *S*/*T* = surgical treatment; and *T*/*T* = targeted therapy.

Fifteen patients (five female and ten male) with OSCC who were receiving pembrolizumab monotherapy as first-line treatment were enrolled in the study (Figure 1). The median age was 69 years (range: 48–89 years). The primary sites of the OSCC were the tongue (*n* = 4, 26.7%), gingiva (*n* = 6, 40.0%), buccal mucosa (*n* = 3, 20.0%), floor of mouth (*n* = 1, 6.67%), and hard palate (*n* = 1, 6.67%). Among the 15 patients, four cases were of recurrent OSCC, nine of unresectable primary OSCC, and two of metastatic OSCC. Thirteen patients had a CPS ≥ 20 while two patients had 1 ≤ CPS ≤ 19.

### 3.2. Efficacy of Pembrolizumab as First-Line Treatment

**Figure 2:**
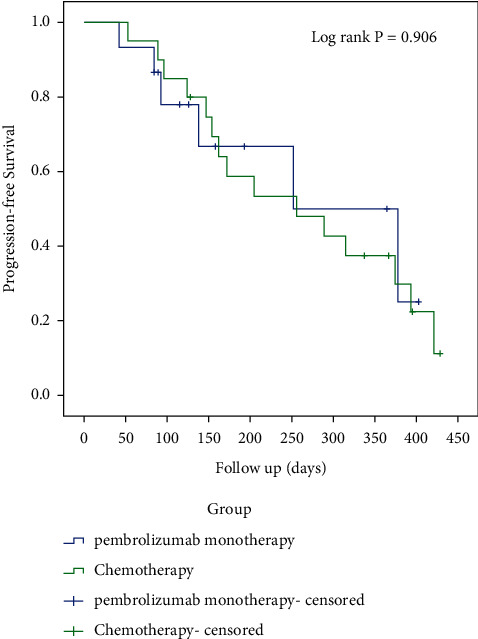
Kaplan–Meier survival curves for progression-free survival of patients with recurrent/unresectable/metastatic oral squamous cell carcinoma treated with pembrolizumab or chemotherapy.

**Figure 3:**
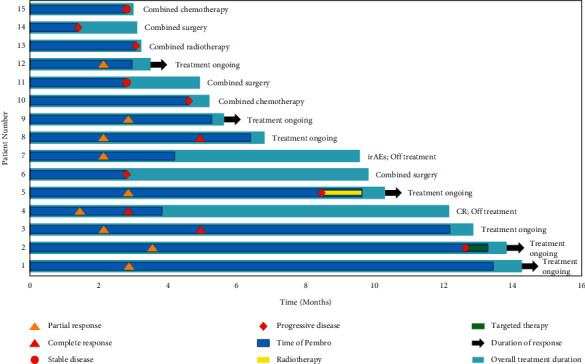
Swimmer plot for a treatment duration of the 15 patients enrolled in our study. CR = complete response; irAEs = immune-related adverse events. The “overall treatment duration” in the swimmer plot referred to the time for pretreatment assessments (including physical examination, imaging study, CPS evaluation, and MDT), pembrolizumab treatment, and subsequent follow-up.

PFS analysis showed that four patients (26.7%) had disease progression at 6-months posttreatment and five patients (33.3%) had disease progression at 1-year posttreatment. The PFS rates were 66.9% and 50.1% at 6 months and 1 year, respectively (Figure 2). Additionally, we found that the PFS rates in the chemotherapy group were 58.7% and 37.3% at 6 months and 1-year posttreatment, respectively. When compared with the chemotherapy group (patients who received conventional chemotherapy without pembrolizumab), the pembrolizumab alone group did not observe significantly improved PFS in the PD-L1 CPS of 1 or more population (*P*=0.906; Figure 2). Nine of 15 patients responded well to single-agent immunotherapy with a median follow-up duration of 9.6 months (range: 3–13.5 months). For the total patients (15/15) with immunotherapy, the median follow-up duration was 6.4 months (range: 2.8–13.5 months). Until the end of the study, the fifteen included patients were all alive and follow-up studies were ongoing. A swimmer plot of outcomes for each of the 15 patients is displayed in Figure 3. The ORR was 60% (9/15). Nine patients started showing positive response to pembrolizumab monotherapy (time to first remission) between two to five cycles (mean: 3.6). The imaging examinations and biopsies after treatment showed that three patients (20%) achieved a complete response, whereas six patients (40%) achieved a partial response (PR). Among them, one patient each transitioned to progressive disease status on the 12th and 18th cycles, respectively. For the patients who responded well to immunotherapy, the mean KPS score after treatment was significantly higher than that before treatment (58.89 ± 13.64 to 85.56 ± 10.14; *P* < 0.001).

### 3.3. Pembrolizumab Treatment-Related Adverse Events

**Figure 4:**
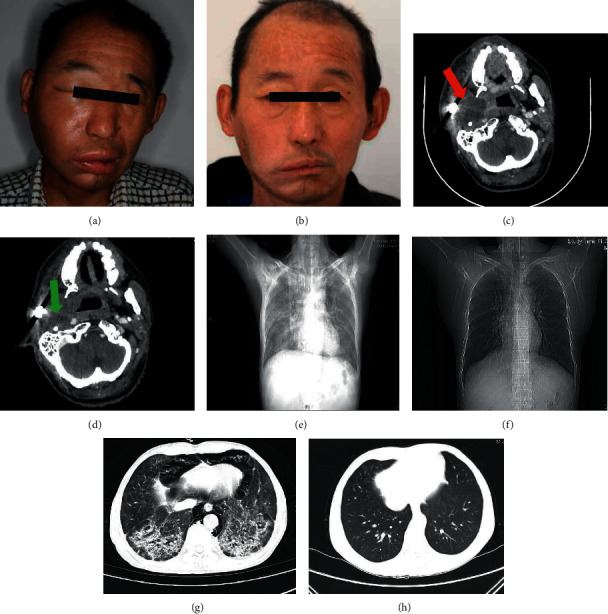
Patient No. 7 experienced tumor recurrence on the right maxilla and an invasion into the pterygoid plate ((a); (c) red arrow). Partial response occurred after four cycles of pembrolizumab treatment ((b); (d) green arrow). Interstitial pneumonia in both lungs was observed after seven cycles of pembrolizumab treatment ((e) chest radiograph; (g) chest computed tomography). The serial images indicate that the immune-related adverse events were controlled following seven days of methylprednisolone treatment ((f) chest radiograph; (h) chest computed tomography).

The adverse reactions observed during the immunotherapy are listed in Table 1. Eight adverse events were observed: four (26.7%) cases of rash and one case each of hypothyroidism, interstitial pneumonia, cheilitis, and cerebral thrombosis (each 6.7%). Among these adverse events, one patient suffered concurrent rash and interstitial pneumonia, whereas another had concurrent rash and cheilitis. The emergency management of severe irAEs should be given attention because it would be life-threatening for patients. In our study, one patient developed breath-holding and coughing and was admitted with grade IV interstitial pneumonia after seven cycles of pembrolizumab. The patient's symptoms were significantly alleviated after 1-week of treatment with the intervention of a high dose of intravenous methylprednisolone and maintenance of airway patency. This patient maintained PR status even after cessation of anti-PD-1 immunotherapy (Figure 4). In another case of severe irAE, the patient had a history of thrombosis and developed symptoms of cerebral thrombosis on the seventh treatment cycle. Despite the permanent cessation of anti-PD-1 immunotherapy, the recurrence of either vascular thrombosis or tumor was not observed during the follow-up of this patient.

## 4. Discussion

Patients with R/U/M OSCC have a poor prognosis, with a median survival of 6–12 months [3, 20]. In recent years, the benefits of immunotherapy in HNSCC have caused a paradigm shift in the treatment of OSCC. On December 11, 2020, pembrolizumab monotherapy was approved by the NMPA of China for treating patients with advanced R/U/M HNSCC with PD-L1 expression (CPS ≥ 20). However, the efficacy and safety of pembrolizumab in Chinese patients with R/U/M HNSCC, especially OSCC, has not been reported adequately due to the relatively short clinical treatment duration. Hence, our case series evaluated the efficacy and safety of pembrolizumab for OSCC treatment in a single-center in China.

Immunotherapy allows the re-establishment of the immune system and is a promising therapy for advanced OSCC. In a KEYNOTE-048 study on HNSCC, 23% (31/133) participants showed an objective response (OR) were reported in the pembrolizumab alone group with PD-L1 expression (CPS ≥ 20) [10]. In our study, the ORR of patients with OSCC was reduced from 60% (9/15) on the fifth cycle to 46.67% (7/15) on the 18^th^ cycle. This change in patients' ORR suggests that a longer follow-up might be more useful for determining pembrolizumab efficacy. O'Donnell et al. [21–23]reported that the acquired immune resistance could lead to tumor progression or recurrence, which may also be related to changes in ORR. Additionally, in 2019, the European Society of Medical Oncology meeting declared that the efficacy of pembrolizumab for HNSCC was much better in the Asian group than in the non-Asian group [10]. In our study, although the time of the first remission of immunotherapy was inconclusive, the OR was observed between two and five cycles (mean: 3.6 cycles). Currently, there are no guidelines describing the time for modifying the treatment regimens for patients receiving single-agent immunotherapy. According to MDT evaluations and our single-center experiences with pembrolizumab, combination therapy may be recommended if patients show no positive response to the single-agent immunotherapy by the fifth cycle.

The management of cancer patients aims to prolong life expectancy and improve QoL. So far, none of the studies have concluded that immunotherapy can significantly improve the PFS and OS of oral cancer patients. In our study, the longest follow-up period for the single-agent immunotherapy was more than 18 cycles (13 months) in patients with good responses. Although there were no PFS benefits by comparing the pembrolizumab group with the chemotherapy group without pembrolizumab in the PD-L1 CPS ≥ 1 population in our study, our patients with pembrolizumab treatment showed a better PFS than those subjected to the chemotherapy regimen in an open-label Phase II trial reported by Chang [24]. Due to the lack of data on the long-termfollow-up of efficacy, it is insufficient to evaluate OS. Indeed, to a certain extent, the QoL of patients with OSCC is considered as important as survival. Advanced oral cancer significantly impacts patients' QoL by adversely influencing their communication and appearance and inducing intractable pain and dysphagia [25–27]. In recent years, immunotherapy has significantly improved QoL for patients with different cancers. In the KEYNOTE-024 study, pembrolizumab was useful for improving or maintaining QoL by relieving symptoms such as cough, chest pain, and dyspnea in lung cancer patients, compared to chemotherapy [28]. In patients responding well to single-agent immunotherapy, the mean KPS score before and after treatment improved from 58.89 ± 13.64 to 85.56 ± 10.14 (*P* < 0.001), suggesting that immunotherapy significantly improved their physical and mental health.

Although immunotherapy has led to a paradigm shift in OSCC treatment, the risk of irAEs in immunotherapy cannot be avoided completely. In our study, we found that mild irAEs (grades 1-2) were predominant compared to severe irAEs (grades 3–5). For patients undergoing immunotherapy, the emergency management of severe irAEs should be established, because they would be life-threatening for patients. Pneumonitis, organizing pneumonia, interstitial pneumonitis, and nonspecific interstitial pneumonia, have been underscored as grade 3–5 irAEs in case reports and clinical studies [29, 30]. Immune-related pneumonitis is a rare but life-threatening adverse reaction that accounts for 35% of PD-1/PD-L1 inhibitor-related deaths [31]. Once PD-1inhibitor-related pneumonitis is recognized, treatment should be immediately stopped and glucocorticoid administration should be considered [32]. In our study, one patient developed severe respiratory failure after seven cycles of pembrolizumab and was diagnosed with immune-related pneumonitis (interstitial pneumonitis). The patient's symptoms were significantly alleviated after administering high doses of intravenous methylprednisolone and maintaining airway patency for one week. In addition, immunotherapy may increase the risk of irAEs, such as thrombosis. Although the correlation between thrombosis and immunotherapy has not been well reported in recent years, it has been reported that checkpoint blockers in patients with cancer could induce accelerated inflammation and lead to an increased risk of thromboembolism and cardiovascular complications [33–36]. In our study, a patient with a history of thrombosis developed symptoms of cerebral thrombosis on the seventh treatment cycle. Despite the permanent cessation of anti-PD-1 immunotherapy, the recurrence of either vascular thrombosis or tumor was not observed during the follow-up period. Our case may contribute to the expanding evidence for the correlation between anti-PD-1-related immunotherapy and the risk of thrombosis.

## 5. Conclusion

MDT is important for single-agent immunotherapy in patients with R/U/M OSCC and should be recommended throughout the treatment period. Existing data lacks a long-termfollow-up to conclusively evaluate the efficacy or OS. However, in our study, patients responding well to anti-PD-1 single-agent immunotherapy showed obvious improvement in QoL. The emergency management of severe irAEs should be established because the risk of irAEs in immunotherapy cannot be avoided completely. Nevertheless, some limitations should be acknowledged in our study. Since our findings came from a single-center study, clinically relevant differences may be found among hospitals. Additionally, a larger sample size should be designed to increase the significance of the results. Overall, we hope that our data can provide a clinical reference for immunotherapy in Chinese patients with R/U/M OSCC.

## Figures and Tables

**Table 1:** Adverse events in 15 patients who were enrolled in our study.

Adverse event	1–2 Grade	3–5 Grade
Rash	4 (26.7%)	0
Hypothyroidism	1 (6.7%)	0
Arrhythmia	0	0
Pneumonitis	0	1 (6.7%)
Stomatitis	1 (6.7%)	0
Liver dysfunction	0	0
Gastroenteritis	0	0
Cerebral thrombosis	0	1 (6.7%)

## Data Availability

No data were used to support this study.
